# Association of angiopoietin-2, C-reactive protein and markers of obesity and insulin resistance with survival outcome in colorectal cancer

**DOI:** 10.1038/sj.bjc.6606005

**Published:** 2010-11-16

**Authors:** E Volkova, J A Willis, J E Wells, B A Robinson, G U Dachs, M J Currie

**Affiliations:** 1 Angiogenesis and Cancer Research Group, Department of Pathology, University of Otago Christchurch, PO Box 4345, Christchurch 8140, New Zealand; 2 Lipid and Diabetes Research Group, Christchurch Hospital, PO Box 4710, Christchurch 8140, New Zealand; 3 Department of Public Health and General Practice, University of Otago Christchurch, PO Box 4345, Christchurch 8140, New Zealand; 4 Oncology Services, Christchurch Hospital, PO Box 4710, Christchurch 8140, New Zealand

**Keywords:** obesity, insulin resistance, tumour angiogenesis, angiopoietin-2, C-reactive protein

## Abstract

**Background:**

This study investigated the relationship of obesity, insulin resistance, inflammation and angiogenesis with cancer progression and survival in a colorectal cancer cohort.

**Methods:**

Clinical and pathological data, along with anthropometric and follow-up data, were collected from 344 consecutive colorectal cancer patients. Serum samples at diagnosis were analysed by immunoassay for adiponectin, C-reactive protein (CRP), vascular endothelial growth factor-A (VEGF-A), angiopoietin-2 (Ang-2), insulin-like growth factor-1 (IGF-1), insulin and C-peptide.

**Results:**

Serum Ang-2 and VEGF-A levels increased with tumour T stage (*P*=0.007 and *P*=0.025, respectively) and N stage (*P*=0.02 and *P*=0.03, respectively), and correlated with CRP levels (*r*=0.43, *P*<0.001 and *r*=0.23, *P*<0.001, respectively). Angiopoietin-2 correlated with C-peptide (*r*=0.14, *P*=0.007) and VEGF-A with IGF-1 in males (*r*=0.25, *P*=0.001). Kaplan–Meier analysis showed that patients with high serum levels of CRP and Ang-2 had significantly reduced survival (both *P*⩽0.001). After adjusting for tumour stage and age, Ang-2 remained a significant predictor of survival. The CRP levels were inversely associated with survival in American Joint Committee on Cancer stage II patients (*P*=0.038), suggesting that CRP could be used to support treatment decisions in this subgroup. Serum markers and anthropometric measures of obesity correlated with each other, but not with survival.

**Conclusion:**

Our study supports the concept that obesity-related inflammation, rather than obesity itself, is associated with colorectal cancer progression and survival. The study confirms serum Ang-2 as a predictive marker for outcome of colorectal cancer.

Colorectal cancer is the third most common cancer in women and the fourth most common cancer in men worldwide (Parkin *et al*, 2005). It is second only to lung cancer as a cause of cancer deaths in New Zealand (Frizelle, 2009), and New Zealand women have both the highest incidence and highest mortality from colorectal cancer in the world (Center *et al*, 2009).

Epidemiological studies have shown that the risk for colorectal cancer development is strongly related to obesity and the metabolic syndrome (Moghaddam *et al*, 2007; Pais *et al*, 2009). The mechanism underlying this association is not completely understood, but obesity-induced insulin resistance, adipokine levels and obesity-related inflammation are all important factors (Sandhu *et al*, 2002; Giovannucci, 2007; Birmingham *et al*, 2009; Gonullu *et al*, 2009), implicating insulin resistance and alterations in the insulin – insulin-like growth factor-1 (IGF-1) axis as the main driving forces (Komninou *et al*, 2003; Giovannucci, 2007). Both insulin and IGF-1 are potent mitogens that promote colorectal cancer cell growth and survival *in vitro* (Komninou *et al*, 2003), and elevated blood levels of IGF-1 and insulin are associated with increased risk of developing colorectal cancer (Komninou *et al*, 2003).

Angiogenesis, the formation of new blood vessels, has a vital function in tumour growth and spread (Rmali *et al*, 2006), and IGF-1 and insulin induce angiogenesis *in vitro* and *in vivo* (Reinmuth *et al*, 2002). Levels of the main angiogenic factors, vascular endothelial growth factor-A (VEGF-A) (Cao *et al*, 2009) and angiopoietin-2 (Ang-2) (Chung *et al*, 2006), are correlated with tumour progression and patient outcome in colorectal cancer.

Despite support for the importance of obesity and metabolic syndrome as risk factors for colorectal cancer development, data are equivocal for their effects on colorectal cancer progression and outcome (Trevisan *et al*, 2001; Dignam *et al*, 2006; Reeves *et al*, 2007; Meyerhardt *et al*, 2008; Moon *et al*, 2008; Wolpin *et al*, 2009). Several studies found worse survival and increased recurrence for patients with insulin resistance or high body mass index (BMI) (Trevisan *et al*, 2001; Dignam *et al*, 2006; Moon *et al*, 2008; Wolpin *et al*, 2009), while other studies reported no significant relationship (Meyerhardt *et al*, 2003; Reeves *et al*, 2007). Obesity influences duration of surgery and post-surgery complications in colorectal cancer patients (Tsujinaka *et al*, 2008; Merkow *et al*, 2009), and alters the response of breast cancer patients to chemotherapy (Litton *et al*, 2008).

In this study, we investigated the relationship of obesity, insulin resistance and inflammation with colorectal cancer progression and survival in a New Zealand colorectal cancer cohort. We propose that obesity-related chronic hyperinsulinemia and insulin resistance promote a pro-inflammatory and pro-angiogenic environment that stimulates tumour growth and metastasis, and leads to poor survival.

## Materials and methods

### Patients

The study cohort comprised consecutive patients undergoing surgery for adenocarcinoma of the colon or upper rectum at Christchurch Hospital between 28 July 1998 and 28 April 2008. All participants had given written informed consent for collection of tumour tissue and blood for research, and samples were obtained after approval from the Cancer Society Tissue Bank (CSTB), Christchurch. The study was approved by the Upper South Ethics Committee (approval number: URB/08/02/006). Stage IV patients (*n*=14) were included, but were highly selected in having low volume metastatic disease, or undergoing colectomy at the time of emergency presentation with obstruction or perforation. All analyses were performed both with this group of stage IV patients included and excluded, and as results were similar, data are presented with stage IV patients included.

Patients were treated according to standard guidelines with pre-operative staging by blood tests for full blood count, liver function tests, chest X-ray and computerised tomography of abdomen and pelvis. In a few cases, the liver was imaged by ultrasound or magnetic resonance imaging (MRI). Patients with rectal cancer also underwent MRI of the pelvis, but were then excluded from this study if they were treated with pre-operative radiation with or without concurrent chemotherapy. The surgical specimens were analysed pathologically by a specialist group of pathologists, although synoptic reporting was only formally introduced in 2005. Staging was by American Joint Committee on Cancer (AJCC) TNM classification (Greene *et al*, 2002). Post-operative adjuvant chemotherapy was offered to patients with nodes involved and also to node-negative patients with adverse features including perforation, vascular or lymphatic invasion and T4 tumours. Either intravenous weekly 5-fluorouracil with leucovorin, or capecitabine, or an oxaliplatin combination was administered. Patients were followed up routinely by the colorectal service at Christchurch Hospital with 6-monthly clinical assessment and blood carcinoembryonic antigen (CEA), with an annual and then 3-yearly colonoscopy, with imaging when indicated on clinical grounds or by CEA rise.

### Sample collection and storage

Blood samples were collected into plain tubes (BD-vacutainer, Franklin Lakes, NJ, USA) from patients on admission to Christchurch Hospital, before colectomy. Blood samples were centrifuged (1800 r.p.m. × 10 min), and the serum aliquoted and stored at −80°C until used in immunoassays.

### Immunoassays

Commercially available Quantikine human ELISA kits (R&D systems, Minneapolis, MN, USA) for adiponectin, high sensitivity-C-reactive protein (CRP), VEGF-A, Ang-2 and IGF-1 and human ELISA kit (Millipore, Billerica, MA, USA) for insulin and C-peptide were used to measure the levels of proteins in patient serum samples. All ELISAs were performed following manufacturers' protocols, with samples assayed in duplicate with appropriate standards as controls.

### Data collection

Demographic and clinical data, along with the pathology report for each patient, were prospectively recorded in the CSTB database. Baseline staging, weight, height, body surface area and BMI were obtained from medical records, together with follow-up information. The BMI was defined as is standard with <18.5 kg m^–2^ underweight; 18.5–24 kg m^–2^ normal; 25–29 kg m^–2^ overweight; ⩾30 kg m^–2^ obese and ⩾35 kg m^–2^ morbidly obese. Diabetes was recorded from the clinical records, but in addition blood glucose levels were checked to disclose previously undiagnosed type 2 diabetes. Follow-up was recorded until 31 August 2009.

### Statistical analysis

Statistical analysis was performed using SPSS version 16 (SPSS Inc., Chicago, IL, USA, 16). Frequency and descriptive statistics were used to describe the cohort. Pearson's product-moment correlations were used to analyse relationships among serum markers, and between serum markers and tumour size, depth and percentage of bowel circumference. Independent-sample *t*-tests were used to compare the levels of serum markers in patients with or without diabetes, lymphatic and vascular invasion, perineural invasion, necrosis or lymph nodes metastasis. One-way analysis of variance and linear test for trend were used to compare the levels of serum markers across tumour stages and grade. Both Kaplan–Meier and Cox regression analyses were performed to analyse patient overall survival. Medians were used to divide continuous data into groups for Kaplan–Meier analysis, with standard cut points for BMI. In Cox regression analysis, tumour stage was analysed as a categorical variable, and age, BMI and serum markers as continuous variables. For the continuous variables, hazard ratios were estimated using the following units: 100 units of VEGF-1, 1000 units of Ang-2, 1 unit of CRP, insulin, C-peptide and BMI, 10 units of IGF-1 and per decade of age. Predictors were entered either on their own, or jointly; stepwise procedures were not used.

## Results

### Colorectal cancer patients

The study cohort of 344 patients included 173 males and 171 females. Individuals ranged in age from 31 to 91 years of age (mean=71, median=73) with 66% of patients aged between 60 and 80 years (Table 1). Only six females were <50 years of age, hence assumed pre-menopausal. Twenty per cent were AJCC stage I, 42% AJCC stage II, 34% stage III and 4% stage IV. Vascular or lymphatic invasion was identified in 101 out of 337 tumours (30%) and perineural invasion in 17 out of 159 tumours (11%), where these were recorded. Twenty-eight individuals (8.1%) had a diagnosis of type 2 diabetes mellitus.

The BMI decreased with advancing age, with no difference by gender (Table 2). Only 2.2% of patients were underweight, with 27.7% normal weight, 45% overweight and 25.1% obese including 6.9% morbidly obese. This distribution reflects the background New Zealand population (Ministry of Health, 2008).

### Clinicopathological and serum factors

Serum levels of the angiogenic factors VEGF-A and Ang-2, and the inflammatory factor CRP, according to clinicopathological features are shown in Table 1. Data for the metabolic factors adiponectin, IGF-1, insulin and C-peptide are available in Table 2.

The VEGF-A levels were significantly higher at more advanced T (tumour) stage (*P*=0.025) and N (nodal) stage (*P*=0.034), but not AJCC stage (*P*=0.07), as well as when lymphatic and vascular invasion was present (*P*=0.02). Angiopoietin-2 levels increased with age (*P*=0.02), more advanced T stage (*P*=0.007) and N stage (*P*=0.02), but did not significantly correlate with AJCC stage (*P*=0.09). Angiopoietin-2 levels were higher when tumour necrosis was present (*P*=0.01), but necrosis data was missing in 48% of cases. The CRP levels increased with tumour AJCC stage (*P*<0.001), T stage (*P*<0.001) and higher grade (*P*=0.004), as well as with increased tumour necrosis (*P*=0.002). Levels of Ang-2 and CRP were significantly higher in women compared with men (*P*=0.001 and *P*<0.001, respectively). Adiponectin levels increased with age (*P*=0.005), were higher in the absence of perineural invasion (*P*=0.03), although data were not available for all patients. Adiponectin levels were higher in women (*P*<0.001) and IGF-1 levels were higher in men (*P*<0.001).

### Surrogate markers of obesity

The anthropometric measure BMI was positively correlated with serum levels of insulin (*r*=0.21, *P*<0.001) and C-peptide (*r*=0.27, *P*<0.001), and negatively correlated with serum levels of adiponectin (*r*=−0.32, *P*<0.001) (Table 3). Insulin showed a positive correlation with C-peptide (*r*=0.63, *P*<0.001), as expected, and IGF-1 was correlated with both insulin (*r*=0.14, *P*=0.01) and C-peptide (*r*=0.14, *P*=0.01). Serum adiponectin showed an inverse correlation with IGF-1, insulin and C-peptide (*r*=−0.21, *P*<0.001; *r*=−0.018, *P*=0.001; *r*=−0.014, *P*=0.01, respectively).

### Obesity, inflammation and angiogenic factors

Serum levels of the angiogenic proteins, Ang-2 and VEGF-A, were correlated (*r*=0.19, *P*<0.001) (Table 3). There was a positive correlation between serum CRP and both VEGF-A (*r*=0.23, *P*<0.0001) and Ang-2 (*r*=0.43, *P*<0.001). Serum levels of Ang-2 and C-peptide were positively correlated (*r*=0.14, *P*=0.007). Serum VEGF-A was positively correlated with IGF-1 in males (*r*=0.25, *P*=0.001), and a similar trend was observed for the whole cohort (*r*=0.10, *P*=0.066; Table 3). Serum CRP levels showed a negative association with IGF-1 (*r*=−0.18, *P*=0.001). Neither VEGF-A nor Ang-2 was associated with BMI or serum adiponectin levels (*P*>0.05).

### Survival analysis

**Figure 1:**
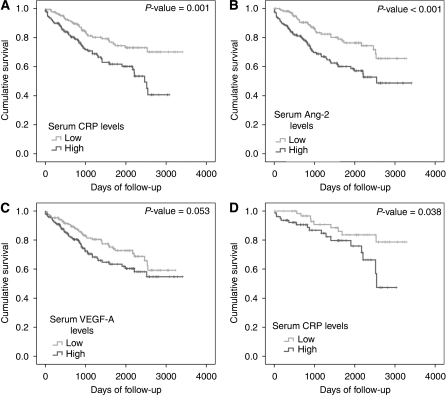
Survival of colorectal cancer patients from surgery to death from any cause by Kaplan–Meier survival analysis. Survival between groups with high and low serum (**A**) CRP, (**B**) Ang-2 and (**C**) VEGF-A. (**D**) Survival of patients with AJCC stage II disease between groups with high and low serum CRP. Median values were used as cut points for high *vs* low values.

During the 10 years of follow-up time, 91 patients died from all causes in the study cohort, with median survival not reached. Eleven of the 14 patients with stage IV disease had died. Kaplan–Meier survival analysis showed that patients with high serum levels of CRP (*P*<0.001; *P*=0.01 excluding stage IV) and Ang-2 (*P*<0.001; *P*=0.002, excluding stage IV) had a significantly worse outcome (Figure 1A and B). High serum VEGF-A was also associated with poorer survival (*P*=0.053; *P*=0.041 excluding stage IV) (Figure 1C). As expected, tumour AJCC stage, T stage, N stage, lymphatic and vascular invasion, and perineural invasion were significantly associated with patient survival (*P*<0.01, data not shown). The BMI did not significantly affect survival (*P*=0.35, data not shown). No association was shown between type 2 diabetes and survival (*n*=28, *P*=0.26).

A separate survival analysis was also completed for patients with AJCC stage II cancer (stages IIA and IIB). The CRP remained a significant predictor of outcome within this group (*P*=0.04, Figure 1D), whereas Ang-2, T stage, lymphatic and vascular invasion, and perineural invasion were not significant predictors of survival in this sub-cohort (data not shown).

Cox regression analysis of individual predictors showed that VEGF-A, Ang-2 and CRP were significant predictors of overall survival for the whole cohort (*P*<0.001; Table 4). These three predictors were further analysed together in a multivariable model, in which VEGF-A and Ang-2 remained significant predictors, whereas CRP was not significant (Table 4, model 1). After adjusting for tumour stage and age, both VEGF-A and CRP lost their predictive value and Ang-2 remained the only significant predictor of survival (Table 4, model 2).

## Discussion

This study demonstrated strong associations of markers of angiogenesis and inflammation with cancer progression and patient survival in a cohort of 344 colorectal cancer patients. Serum levels of Ang-2 emerged as strongly predictive of overall survival in our multivariable survival analysis. Angiopoietin-2 regulates tumour angiogenesis (Ahmad *et al*, 2001a, 2001b; Sarraf-Yazdi *et al*, 2008), and increased levels of tumour Ang-2 are associated with more aggressive, angiogenic CRC tumours (Chung *et al*, 2006). The positive correlations observed between serum Ang-2 and serum C-peptide (a stable marker of circulating insulin levels), and between VEGF-A and IGF-1 in males, may implicate insulin and IGF-1 in promoting a systemic pro-angiogenic environment, and potentially increasing tumour angiogenesis.

Prevalence of insulin resistance and type 2 diabetes varies markedly by age, as well as ethnicity. In individuals aged >60 years, the prevalence of diagnosed diabetes in New Zealand is 9.5% for Europeans, and 21.0% and 24.5% for Māori and Pacific Island people, respectively (Ministry of Health, 2007). The ethnic breakdown of this clinical cohort reflects that of the Canterbury background population, which is predominantly European (77.4% European, 7.2% Māori) (Morrin *et al*, 2005; Census, 2006). The prevalence of diagnosed type 2 diabetes was 8.1% in this colorectal cancer cohort, reflecting that of the background population. Our study did not find a direct relationship between type 2 diabetes and colorectal cancer, but the total number with diabetes was relatively small.

It is now well established that obesity is characterised by chronic inflammation (Greenberg and Obin, 2006; Park *et al*, 2010), with associated increases of CRP, interleukin-6 (IL-6) and plasminogen activator inhibitor (Dandona *et al*, 2004). Recent *in vivo* data in dietary and genetically obese mouse models demonstrated obesity-related liver inflammation and subsequent release of IL-6 and TNF*α* (Park *et al*, 2010). Our clinical data supports a significant association between Ang-2 and inflammation, via the acute phase inflammatory protein, CRP. This association is supported by *in vitro* data (Bello *et al*, 2008; Turu *et al*, 2008; Porta *et al*, 2009) and recent data from an Ang-2 knock-out mouse model, where lack of Ang-2 reduced inflammatory bowel disease (Ganta *et al*, 2010). However, CRP may also mediate inhibition of angiogenesis, as shown *in vitro* (Yang *et al*, 2005). Our clinical findings suggest that the influence of inflammation on colorectal cancer progression and outcome may involve Ang-2-mediated pathways. To support these observations, markers of angiogenesis are being investigated in tumour samples from this patient cohort.

In our study, serum levels of Ang-2 were a stronger predictor of survival than serum levels of VEGF-A, the principal angiogenic factor associated with poor outcome in colorectal cancer (Cao *et al*, 2009). Circulating levels of Ang-2 have been shown to correlate with poor patient survival in other cancers including melanoma and lung cancer (Park *et al*, 2007; Helfrich *et al*, 2009), and patients with metastatic colorectal cancer have higher levels of serum Ang-2 than healthy controls (Goede *et al*, 2010). Angiopoietin-2 regulates vascular remodelling and endothelial responsiveness to pro-inflammatory cytokines (Fiedler *et al*, 2006). In addition, recent *in vitro* and *in vivo* studies have demonstrated that Ang-2 acts as a chemoattractant for pro-angiogenic Tie2-expressing monocyte/macrophages (TEM), and stimulates TEM to express tumour-promoting factors. Mice with Ang-2 over-expressed in tumour vasculature had high serum Ang-2 levels, increased TEM infiltration of tumours and an increased number of tumour microvessels with immature phenotype (Murdoch *et al*, 2007; Coffelt *et al*, 2010). Thus, high levels of Ang-2 may impact patient survival by facilitating tumour vascular disruption, and by skewing tumour-infiltrating leukocytes towards an alternatively activated (M2) phenotype that promotes tumour angiogenesis and progression.

Experimental and epidemiological studies support the concept that chronic inflammation has cancer-promoting properties (Mantovani *et al*, 2008; Porta *et al*, 2009). In our study, elevated serum CRP levels were positively associated with markers of more advanced disease and worse overall patient survival, consistent with other studies (Nozoe *et al*, 1998, 2008). In these studies, CRP levels above the upper limit of normal of 5 mg l^–1^ (Nozoe *et al*, 2008) or 8 mg l^–1^ (Nozoe *et al*, 1998) were considered elevated. However, in cardiovascular disease, CRP is an established risk factor at levels as low as 0.49 mg l^–1^ (Ridker *et al*, 2002), and no such threshold has yet been determined for cancer. Therefore, in our study, CRP levels were treated as a continuous variable, and median (4.1 mg l^–1^) was used as a cut point between low and high levels of CRP.

The CRP was the only significant predictor of overall survival in our sub-cohort of 144 AJCC stage II patients in a multivariable analysis. While clinical factors are currently used to identify stage II patients who have a poor prognosis and hence require adjuvant chemotherapy, a predictive serum biomarker would be of direct clinical utility. Although Nozoe *et al* (2008) reported CRP to be prognostic in a group of 116 patients with all Dukes stages, only 34 had Dukes B disease. Our data suggest that CRP could be used to support decisions about adjuvant chemotherapy, but would need further testing in stage II patients.

Associations between CRP and other surrogate markers of obesity were not significant in this study, although this link is supported in the literature (Koukourakis *et al*, 2009; Nguyen *et al*, 2009). A limitation of our study may be the decision to measure CRP at diagnosis, which may have obscured the contribution from obesity, as inflammation within the primary tumour may have been the main contributor to high serum CRP. This is supported by the increase in CRP with T stage. A large study in healthy adults across the weight spectrum in the United States, found a direct correlation between serum CRP levels and increasing BMI (Nguyen *et al*, 2009). A similar correlation was observed in cancer patients with no detectable tumour, but was lost in cancer patients with evident cancer burden (Koukourakis *et al*, 2009). Together with our data, this suggests that CRP from inflammation in advanced cancer may obscure that from obesity-related inflammation.

None of the markers of obesity (BMI and serum markers) showed an association with tumour progression or patient survival, for the whole cohort, or by gender. The relationship between obesity and patient survival remains equivocal. In a study of over 4000 colorectal cancer patients, morbidly obese patients were 40% more likely to have a recurrence or secondary tumour, and 30% more likely to die, compared with patients with normal BMI (Dignam *et al*, 2006). In contrast, a similar sized study showed no difference in overall, disease-free (DFS) or recurrent-free survival across all BMI groups (Meyerhardt *et al*, 2003), except that obese women younger than 50 years of age had a worse outcome compared with women with normal BMI. Our cohort were an older population, with 96% of patients over 50 years of age and only six women <50 years old. Our study did not determine waist circumference, and a recent, smaller study (Haydon *et al*, 2006) found that waist circumference, but not BMI, was associated with survival. A subsequent study by Meyerhardt found that morbidly obese patients had decreased DFS, but not overall survival (Meyerhardt *et al*, 2008). Only 6.9% of patients in our study were morbidly obese, and they could not be analysed separately. The distribution of BMI categories in our study (25.1% >BMI 30) compared well with other studies (17.5–34.0% >30 BMI) (Dignam *et al*, 2006; Reeves *et al*, 2007; Meyerhardt *et al*, 2008). Hence, current data suggest that severe obesity, rather than a continuum of BMI, impacts negatively on survival from colorectal cancer.

Owing to the proven unreliability of BMI as a marker of obesity, our study sought to define surrogate serum markers of obesity. While total serum levels of adiponectin and IGF-1 were measured, our assay system was unable to distinguish high molecular weight multimers of adiponectin, which represent the most biologically active form (Kadowaki *et al*, 2006), and may have better predictive value. In addition, the IGF-binding proteins, which regulate bioavailable levels of IGF-1 in circulation (Fuchs *et al*, 2008), were not measured. Despite these limitations, our study demonstrated a consistent and significant relationship among the serum markers of obesity measured (insulin, C-peptide, IGF-1, adiponectin, BMI), supporting the conclusion of a limited relationship between obesity and colorectal cancer survival. We, therefore, propose that the influence of obesity on tumour progression and survival in colorectal cancer may be due to obesity-related inflammation, rather than factors associated with obesity *per se*.

We have reported serum markers of obesity, inflammation and angiogenesis at diagnosis of colorectal cancer, and correlated them with clinicopathological variables and with outcome. We did not confirm a worse outcome from diagnosis for obese patients, or for type 2 diabetes, although this conclusion may be limited by small numbers. Highly sensitive CRP, a marker of inflammation, was associated with survival, increased with tumour stage and may have reflected inflammation in the tumour as well as that due to obesity. We have established the value of the pro-angiogenic factor Ang-2 in serum to predict survival. We have shown an association between obesity, inflammation, angiogenesis and outcome, but not demonstrated a role of the insulin-IGF-1 axis. However, the possible effects of obesity and insulin-IGF-1 on response to chemotherapy treatment warrant further study.

## Figures and Tables

**Table 1:** Serum angiogenic and inflammatory factors according to clinicopathological features in colorectal cancer patients

		**CRP (*μ*g ml^–1^)**			**VEGF-A (pg ml^–1^)**			**Ang-2 (pg ml^–1^)**		
	**Total *N***	**Mean**	**Standard deviation**	***P*-value**	**Mean**	**Standard deviation**	***P*-value**	**Mean**	**Standard deviation**	***P*-value**
*Gender (N total*=*344)*										
Female	171	5.44	3.05	<0.001^a^	432	375	0.084^a^	3050	1501	0.001^a^
Male	173	4.02	3.07		364	345		2536	1311	
										
*Age groups (N total*=*344)*										
31–50	13	3.52	2.42		275	210		2591	2413	
51–60	28	4.53	3.14	0.030^b^	430	417	0.732^b^	2061	854	0.004^b^
61–70	102	4.12	3.05	0.026^c^	398	370	0.425^c^	2600	1306	0.022^c^
71–80	146	4.99	3.12		409	343		2928	1482	
81–91	55	5.55	3.28		379	394		3206	1287	
										
*AJCC (N total*=*343)*										
I	69	3.20	2.57		367	332		2637	1501	
IIA	115	5.00	3.14		323	263		2674	1301	
IIB	29	5.50	3.48	<0.001^b^	495	450	0.021^b^	2972	1189	0.258^b^
IIIA	9	4.45	3.42	<0.001^c^	309	181	0.066^c^	2649	1344	0.093^c^
IIIB	71	4.85	3.07		441	430		2766	1357	
IIIC	36	5.05	3.16		537	402		3331	1970	
IV	14	7.38	2.08		459	469		3057	1207	
										
*T stage (N total*=*342)*										
T1	28	3.18	2.67		246	267		2369	1064	
T2	59	3.71	2.77	<0.001^b^	440	359	0.064^b^	2776	1583	0.007^b^
T3	182	4.92	3.08	<0.001^c^	388	362	0.025^c^	2664	1278	0.007^c^
T4	73	5.64	3.38		447	383		3276	1672	
										
*N stage (N total*=*337)*										
N0	214	4.54	3.17		361	320		2709	1357	
N1	79	4.86	3.01	0.181^b^	432	439	0.061^b^	2789	1325	0.069^b^
N2	44	5.48	3.14	0.07^c^	488	387	0.034^c^	3267	1872	0.021^c^
										
*Grade (N total*=*245)*										
1	8	2.69	1.89	0.001^b^	566	412	0.094^b^	2552	865	0.087^b^
2	176	4.46	3.03	0.004^c^	376	358	0.335^c^	2719	1412	0.245^c^
3	58	5.91	3.33		467	403		3194	1716	
										
*Lymph/vascular invasion (N total*=*337)*										
No	236	4.49	3.01	0.102^a^	354	301	0.016^a^	2685	1268	<0.001^a^
Yes	101	5.14	3.42		469	431		3053	1754	
										
*Perineural invasion (N total*=*159)*										
No	135	4.50	3.29	0.331^a^	461	366	0.487^a^	2877	1522	0.805^a^
Yes	17	5.32	3.14		396	330		2977	1869	
										
*Necrosis (N total*=*180)*										
No	142	4.34	3.01	0.002^a^	357	354	0.559^a^	2536	1101	0.012^a^
Yes	38	6.12	3.35		395	351		3084	1463	
										
*Lymphocytic infiltrate (N total*=*267)*										
No	92	4.69	3.45		428	343		2738	1365	
1	112	5.02	2.99	0.270^b^	360	321	0.192^b^	2728	1340	0.589^b^
2	49	4.21	3.02	0.153^c^	327	263	0.126^c^	2610	1411	0.176^c^
3	14	3.64	2.49		296	300		2246	701	

Abbreviations: AJCC=American Joint Committee on Cancer; Ang-2=angiopoietin-2; ANOVA=analysis of variance; CRP=C-reactive protein; VEGF-A=vascular endothelial growth factor-A.

a Independent-samples *t*-test.

b One-way ANOVA.

c Test for linear trend.

**Table 2:** Obesity-related factors according to clinicopathological features in colorectal cancers patients

		**Adiponectin (ng ml^–1^)**			**IGF-1 (ng ml^–1^)**			**Insulin (*μ*U ml^–1^)**			**C-peptide (*μ*g ml^–1^)**			**BMI**		
	**Total *N***	**Mean**	**Standard deviation**	***P*-value**	**Mean**	**Standard deviation**	***P*-value**	**Mean**	**Standard deviation**	***P*-value**	**Mean**	**Standard deviation**	***P*-value**	**Mean**	**Standard deviation**	***P*-value**
*Gender (N total*=*344)*																
Female	171	10213	6514	<0.001^a^	82.04	30.56	<0.001^a^	12.94	23.46	0.106^a^	4.41	3.51	0.128^a^	27.45	6.05	0.745^a^
Male	173	7037	5107		104.15	36.83		17.07	23.82		5.01	3.82		27.64	4.64	
																
*Age groups (N total*=*344)*																
31–50	13	6668	5822		99.76	41.46		24.18	32.41		4.26	2.96		28.87	6.03	
51–60	28	6989	4474	0.003^b^	96.9	28.78	0.076^b^	17.7	20.69	0.612^b^	4.27	3.68	0.444^b^	29.36	4.12	0.003^b^
61–70	102	7812	5739	0.005^c^	99.35	31.06	0.095^c^	14.45	21.99	0.108^c^	4.35	3.39	0.231^c^	28.57	6.07	0.012^c^
71–80	146	8671	5486		91.21	36.09		14.77	24.94		4.81	3.84		27.2	5.13	
81–91	55	11250	7884		83.39	41.76		13.2	22.74		5.42	3.88		25.44	4.33	
																
*AJCC (N total*=*343)*																
I	69	9965	6382		94.96	37.14		13	16.35		4.83	3.24		27.37	7.36	
IIA	115	8588	5922		93.16	37.25		14.41	21.69		4.39	3.47		27.92	4.64	
IIB	29	7289	5097	0.148^b^	102.91	31.73	0.699^b^	19.57	30.36	0.873^b^	5.98	4.38	0.586^b^	27.71	5.48	0.956^b^
IIIA	9	6945	6117	0.082^c^	86.4	19.76	0.208^c^	13.1	18.17	0.93^c^	4.06	3.12	0.775^c^	26.7	4.68	0.892^c^
IIIB	71	8322	5607		90.72	33.58		17.3	30.78		4.7	3.84		27.28	5.19	
IIIC	36	9338	7687		92.93	37.94		14.18	23.55		4.71	4.32		27.04	3.84	
IV	14	5672	3448		84.42	33.62		13.22	19.87		4.7	3.69		28.35	5.29	
																
*T stage (N total*=*342)*																
T1	28	9455	6342		94.68	35.45		17.08	17.81		5.43	3.83		28.3	5.49	
T2	59	8927	6292	0.811^b^	93.52	35.73	0.989^b^	11.16	16.02	0.534^b^	4.35	2.77	0.654^b^	27.05	7.71	0.443^b^
T3	182	8583	6091	0.336^c^	92.79	36.14	0.735^c^	15.36	24.51	0.842^c^	4.73	3.81	0.463^c^	27.9	4.7	0.374^c^
T4	73	8243	5791		92.18	34.01		16.77	28.75		4.69	3.99		26.89	4.84	
																
*N stage (N total*=*337)*																
N0	214	8918	5997	0.449^b^	94.63	36.66	0.577^b^	14.62	21.52	0.703^b^	4.74	3.56	0.985^b^	27.73	5.75	0.736^b^
N1	79	7907	5467	0.82^c^	90	32.53	0.577^c^	16.84	30	0.776^c^	4.66	3.9	0.925^c^	27.29	5.32	0.539^c^
N2	44	8666	7258		91.34	35.39		13.47	21.75		4.68	4.07		27.16	3.77	
																
*Grade (N total*=*245)*																
1	8	10699	8464	0.563^b^	89.7	25.03	0.207^b^	10.39	13.76	0.231^b^	3.62	2.14	0.463^b^	23.97	5.18	0.071^b^
2	176	9105	5645	0.998^c^	96.81	36.67	0.812^c^	17.11	26.21	0.691^c^	4.92	3.94	0.394^c^	27.73	5.6	0.249^c^
3	58	8670	5676		87.13	39.43		11.57	14.86		4.57	2.95		26.84	4.33	
																
*Lymph/vascular invasion (N total*=*337)*																
No	236	8724	6099	0.938^a^	94.21	34.52	0.373^a^	14.08	21.87	0.505^a^	4.69	3.54	0.977^a^	27.56	5.55	0.71^a^
Yes	101	8668	6048		90.44	37.71		15.92	26		4.68	3.96		27.31	4.95	
																
*Perineural invasion (N total*=*159)*																
No	135	7023	5870	0.027^a^	94.69	32.84	0.609^a^	13.05	22.93	0.94^a^	4.18	3.34	0.928^a^	28.06	5.44	0.887^a^
Yes	17	5099	2755		90.37	32.46		12.61	18.35		4.1	2.59		27.85	6.76	
																
*Necrosis (N total*=*180)*																
No	142	9645	5574	0.351^a^	94.38	38.89	0.77^a^	16.26	22.53	0.915^a^	5.08	3.91	0.81^a^	27.64	4.82	0.918^a^
Yes	38	8707	5152		92.34	34.39		15.81	27.05		4.91	4.19		27.54	5.81	
																
*Lymphocytic infiltrate (N total*=*267)*																
No	92	7527	4346		95.01	37.05	0.617^b^	14.06	25.53	0.62^b^	4.03	3.13	0.234^b^	27.46	4.7	
1	112	9838	5737	0.014^b^	91.17	33.96	0.923^c^	16.37	24.45	0.605^c^	5.06	3.93	0.907^c^	27.27	5.85	0.928^b^
2	49	9638	6135	0.074^c^	87.69	33.65		18.19	26.79		4.72	3.82		27.05	4.94	0.521^c^
3	14	10497	8107		97.15	32.16		9.67	16.24		4.27	3.26		26.46	5.43	

Abbreviations: AJCC=American Joint Committee on Cancer; ANOVA=analysis of variance; BMI=body mass index; IGF-1=insulin-like growth factor-1.

a Independent-samples *t*-test.

b One-way ANOVA.

c Test for linear trend.

**Table 3:** Associations of angiogenic, inflammation and obesity-related factors in colorectal cancer patients

	**VEGF-A (*N*=344)**	**Ang-2 (*N*=344)**	**Adiponectin (*N*=344)**	**CRP (*N*=344)**	**IGF-1 (*N*=344)**	**Insulin (*N*=344)**	**C-peptide (*N*=344)**
*Ang-2 (N*=*344)*							
Pearson's correlation	0.19						
*P*-value	0.000						
							
*Adiponectin (N*=*344)*							
Pearson's correlation	−0.04	0.05					
*P*-value	0.441	0.314					
							
*CRP (N*=*344)*							
Pearson's correlation	0.23	0.43	−0.02				
*P*-value	0.000	0.000	0.788				
							
*IGF-1 (N*=*344)*							
Pearson's correlation	0.10	−0.01	−0.21	−0.18			
*P*-value	0.066	0.894	0.000	0.001			
							
*Insulin (N*=*344)*							
Pearson's correlation	0.03	0.02	−0.18	−0.08	0.14		
*P*-value	0.587	0.679	0.001	0.142	0.010		
							
*C-peptide (N*=*344)*							
Pearson's correlation	0.02	0.14	−0.14	−0.02	0.14	0.63	
*P*-value	0.738	0.007	0.010	0.690	0.010	0.000	
							
*BMI (N*=*318)*							
Pearson's correlation	0.04	−0.03	−0.32	0.07	0.09	0.21	0.27
*P*-value	0.448	0.542	0.000	0.241	0.105	0.000	0.000

Abbreviations: Ang-2=angiopoietin-2; BMI=body mass index; CRP=C-reactive protein; IGF-1=insulin-like growth factor-1; VEGF-A=vascular endothelial growth factor-A.

**Table 4:** Cox regression survival analyses

	**Hazard ratio^a^**	**95% CI**	**Total *P*-value**
*Individual predictors*			
VEGF-A	1.09	(1.04–1.15)	<0.001
Ang-2	1.27	(1.13–1.42)	<0.001
CRP	1.13	(1.06–1.21)	<0.001
Age	1.37	(1.08–1.75)	0.010
Insulin	1.00	(0.99–1.01)	0.924
C-peptide	1.02	(0.96–1.07)	0.590
Adiponectin	1.00	(0.97–1.04)	0.937
IGF-1	1.00	(0.94–1.06)	0.982
*BMI groups:*			
Underweight	1.94	(0.59–6.45)	
Normal	1.00		0.371
Overweight	0.81	(0.49–1.35)	
Obese	0.74	(0.40–1.33)	
*AJCC stages:*			
Stage I	1.00		
Stage II	1.41	(0.64–3.12)	<0.001
Stage III	4.56	(2.15–9.68)	
Stage IV	15.29	(6.10–38.38)	
			
*Multivariable model 1*			
VEGF-A	1.07	(1.01–1.12)	0.018
Ang-2	1.17	(1.02–1.34)	0.024
CRP	1.07	(0.10–1.15)	0.067
			
*Multivariable model 2*			
*AJCC stages:*			
Stage I	1.00		
Stage II	1.40	(0.62–3.14)	<0.001
Stage III	4.30	(1.99–9.29)	
Stage IV	17.57	(6.53–47.28)	
Age	1.41	(1.10–1.80)	0.006
VEGF-A	1.04	(0.99–1.09)	0.136
Ang-2	1.23	(1.06–1.42)	0.006
CRP	1.00	(0.93–1.09)	0.956

Abbreviations: AJCC=American Joint Committee on Cancer; Ang-2=angiopoietin-2; BMI=body mass index; CI=confidence interval; CRP=C-reactive protein; IGF-1=insulin-like growth factor-1; VEGF-A=vascular endothelial growth factor-A.

a Change in Hazard ratio for continuous variables was estimated using the following units: 100 units VEGF-1, 1000 units Ang-2, 1 unit CRP, insulin and C-peptide, 10 units IGF-1 and per decade of age.
